# Methods to support evidence-informed decision-making in the midst of COVID-19: creation and evolution of a rapid review service from the National Collaborating Centre for Methods and Tools

**DOI:** 10.1186/s12874-021-01436-1

**Published:** 2021-10-27

**Authors:** Sarah E. Neil-Sztramko, Emily Belita, Robyn L. Traynor, Emily Clark, Leah Hagerman, Maureen Dobbins

**Affiliations:** 1 National Collaborating Centre for Methods and Tools, McMaster University, 175 Longwood Rd S, Suite 210a, ON L8P 0A1 Hamilton, Canada; 2 Department of Health Research Methods, Evidence & Impact, McMaster University, 2C Area, 1280 Main St W, ON L8S 4K1 Hamilton, Canada; 3 School of Nursing, McMaster University, 2J20, 1280 Main St W, ON L8S 4K1 Hamilton, Canada; 4 Department of Community Health & Epidemiology, Centre for Clinical Research, Dalhousie University, 5790 University Ave, Halifax B3H 1V7 NS, Canada

**Keywords:** Rapid review, Evidence synthesis, COVID-19, Knowledge translation, Evidence-informed decision-making, Public health

## Abstract

**Background:**

The COVID-19 public health crisis has produced an immense and quickly evolving body of evidence. This research speed and volume, along with variability in quality, could overwhelm public health decision-makers striving to make timely decisions based on the best available evidence. In response to this challenge, the National Collaborating Centre for Methods and Tools developed a Rapid Evidence Service, building on internationally accepted rapid review methodologies, to address priority COVID-19 public health questions.

**Results:**

Each week, the Rapid Evidence Service team receives requests from public health decision-makers, prioritizes questions received, and frames the prioritized topics into searchable questions. We develop and conduct a comprehensive search strategy and critically appraise all relevant evidence using validated tools. We synthesize the findings into a final report that includes key messages, with a rating of the certainty of the evidence using GRADE, as well as an overview of evidence and remaining knowledge gaps. Rapid reviews are typically completed and disseminated within two weeks. From May 2020 to July 21, 2021, we have answered more than 31 distinct questions and completed 32 updates as new evidence emerged. Reviews receive an average of 213 downloads per week, with some reaching over 7700. To date reviews have been accessed and cited around the world, and a more fulsome evaluation of impact on decision-making is planned.

**Conclusions:**

The development, evolution, and lessons learned from our process, presented here, provides a real-world example of how review-level evidence can be made available – rapidly and rigorously, and in response to decision-makers’ needs – during an unprecedented public health crisis.

**Supplementary Information:**

The online version contains supplementary material available at 10.1186/s12874-021-01436-1.

## Background

Coronavirus disease 2019 (COVID-19) is an urgent public health crisis requiring prompt decision-making due to rapidly evolving policy and practice needs. Public health decision-makers are always challenged with integrating research into decision-making [1]. This has been further exacerbated with an explosion of COVID-19 evidence due to the increased availability of pre-prints that have not yet undergone peer review, and as publishers expedite steps in the peer-review process to make evidence available in a timely manner [2, 3]. An analysis of Web of Science and Scopus found 23,634 COVID-19-related documents from January-June 2020 [4]; this, compared to a PubMed search revealing 28,300 cardiovascular disease-related publications in all of 2019.

Knowledge syntheses (e.g. best practice guidelines, systematic reviews) represent the highest levels of research evidence [5], summarizing and interpreting results of individual studies and contextualizing them within a larger body of knowledge [6]. An up-to-date guideline based on high-quality systematic reviews is considered the best source of evidence for decision-making [7]. The time to conduct a full systematic review and guideline (> 1–2 years [8]) vastly exceeds the time available to make urgent decisions during public health crises [8]. As a result, several global evidence synthesis organizations [9–11] pivoted to producing COVID-19-related rapid reviews (RR). RRs can be defined as “a form of knowledge synthesis that accelerates the process of conducting a traditional systematic review through streamlining or omitting a variety of methods to produce evidence in a resource-efficient manner” [12]. A number of different methodological approaches to conducting a RR exist in order to ‘streamline’ the approach, including limiting the number of databases or timeframe searched, using only a single reviewer for screening, data extraction and/or critical appraisal, or omitting steps such as critical appraisal, meta-analysis, and fulsome write-up [13]. As RRs may have a greater likelihood of bias due to expedited processes, transparency in method is important, with explicit identification of departures from systematic review methods [14–16]. A systematic and rigorous process should be maintained with respect to searching, study selection, data extraction, and quality assessment [17]. The production of high-quality syntheses, including critical appraisal of included studies, is particularly important in the current COVID-19 “infodemic” [18, 19].

The six National Collaborating Centres for Public Health were created by the Public Health Agency of Canada (PHAC) in 2005 to strengthen public health in response to the 2003 Severe Acute Respiratory Syndrome (SARS) epidemic [20]. They exist to support the timely use of scientific evidence and other knowledge in public health practice, programs, and policies. The National Collaborating Centre for Methods and Tools’ (NCCMT) vision is for stronger public health, driven by the best-available evidence, to improve the health and well-being of Canadians [21]. The NCCMT acts as an evidence intermediary, curating trustworthy scientific evidence and building capacity for individuals and organizations in public health to find, interpret, adapt, and implement evidence. Ongoing collaboration with a broad network ensures NCCMT’s agility and responsiveness to evolving public health needs, which have been vital in supporting the pandemic response.

As COVID-19 unfolded, the NCCMT heard from public health decision-makers at all levels of government (local, regional, provincial/territorial, federal) about the lack of time and human resources to find answers to key questions. These decision-makers ranged from managers responsible for front line public health staff at local health departments, to members of federal government advisory committees responsible for key recommendations and policies related to various aspects of the pandemic response. To address this need – and with encouragement from our funder to focus and reallocate resources – the NCCMT pivoted to completing RRs within 5–10 business days based on priority questions from public health decision-makers.

In reviewing NCCMT’s established RR protocol [22], the team realized modifications were required given the emergence of a unique evidence ecosystem for COVID-19. The expanded evidence-base [23], new COVID-19-dedicated databases, and increased use of preprint servers [24] complicated established searching, screening, and quality appraisal processes. This presented new challenges for conducting reviews that were timely, efficient, and rigorous.

**Fig. 1:**
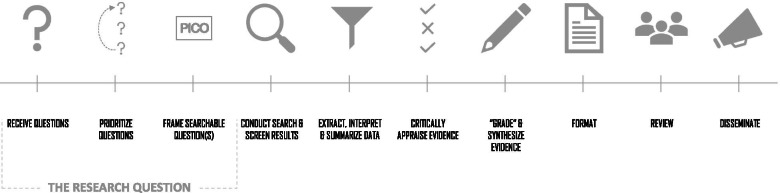
Overview of NCCMT’s Rapid Evidence Service process

Here we describe in detail the methods the NCCMT has used to conduct RRs as part of our Rapid Evidence Service (RES), including how these have evolved to ensure feasibility, accuracy, and efficiency as the evidence landscape changed. Our process (Fig. 1) may be used as a guide for other organizations conducting RRs, in response to COVID-19 and other emerging public health issues, now and in the future.

## Evolution of the RES

The initial protocol built upon the five steps in the NCCMT’s RR Guidebook [22]: defining the research question, searching for, critically appraising, and synthesizing evidence, and assessing applicability and transferability. In the early phases of the pandemic, our team answered questions in as few as five business days. As needs evolved and evidence volume grew, the time to complete reviews was approximately 7–10 business days, and up to three or four weeks for complex, multi-question topics with a large amount of available evidence.

The RES requires a team with methodological and organizational expertise (Table 1) in: conducting rigorous systematic reviews; articulating answerable research questions; creating a search strategy and searching databases, including COVID-19 specific databases; critically appraising different study designs; synthesizing evidence for key findings and actionable messages; and using Grading of Recommendations, Assessment, Development and Evaluation (GRADE) methodology to rate certainty of evidence [25].

**Table 1:** Overview of NCCMT Rapid Evidence Service team and key responsibilities

Role (number of staff)	Responsibilities
NCCMT Scientific Director (1)	• Approves question prioritization • Approves completed reviews • Advises on methodological decisions
NCCMT Operational Lead (1)	• Approves resource allocation • Reviews weekly plan and helps identify potential conflicts
RES Scientific Lead (1)	• Makes decisions on methodological approach to reviews • Approves deviations from standard protocol • Conducts internal peer review of all rapid reviews
Rapid Review Lead (3)	• Oversees entire process, per review • Defines question(s) and PICO(s) • Identifies search terms • Completes full text review of results and makes final decisions on study inclusion • Decides how best to organize each review • GRADEs the evidence • Writes the Executive Summary and key messages for dissemination
RES Coordinator (1)	• Triages question requests, logs questions for team consideration, and consults with RES Scientific Lead, as needed • Documents decisions from weekly team meetings • Contributes to question definition and PICO(s) • Liaises with Rapid Review Lead(s) and staff to coordinate each review • Assigns review teams and develops schedule • Ensures protocol followed • Implements Rapid Review Lead(s)/RES Scientific Lead decision(s) (e.g., review framing and presentation) • Supports staff questions in search, appraise, and study summary stages, consulting RES Scientific Lead, as needed • Facilitates dissemination of reviews • Contributes to review support stages, as needed
Rapid Review Search Lead (1)	• Conducts initial search of key sources for similar recent reviews • Works with RES Scientific Lead and Rapid Review Lead(s) to refine search strategy or inclusion criteria, as needed • Assigns search tasks to Rapid Review Search Staff, fields questions
Rapid Review Search Staff (1–2)	• Searches databases and tracks results • Completes title and abstract level screening
Rapid Review Internal Staff Support (3–4)	• Uses existing template to build rapid review document • Contributes to data extraction and critical appraisal of included studies • Formats final review document • Posts to RES web page and rapid review repository (in progress and complete) • Posts social media content
Rapid Review External Contractor Support (3–4)	• Contributes to data extraction and critical appraisal of included studies
Abbreviations: GRADE: Grading of Recommendations, Assessment, Development and Evaluation; NCCMT: National Collaborating Centre for Methods and Tools; PICO: Population, Intervention, Comparison, Outcome; RES: Rapid Evidence Service *Note*: Some staff contribute to more than one role, e.g., the RES Scientific Lead also acts as a Rapid Review Lead, search staff may contribute as internal support staff, etc.	

### Defining the research question

Limited resources were initially allocated to the RES; questions were restricted to two per week. Questions were prioritized from a list of urgent topics by the NCCMT Scientific Director, Operational Lead, and RES Scientific Lead, primarily based on team expertise and capacity. As demand grew, 60 % of NCCMT staff resources were re-allocated to the RES. Questions initially came from PHAC. As awareness of the service grew, requests came from decision-makers at all levels across Canada and indirectly through involvement in the COVID-19 Evidence Network to support Decision-Makers (COVID-END) [26]. As requests increased, the RES coordination role was formalized to exclusively plan workflow, assign staff tasks, and monitor completion. Requests were formally received through a direct email to the NCCMT’s central email account; through a key contact at our funder, the PHAC, who coordinates evidence synthesis requests from a number of decision-makers and committees within the agency; and through the COVID-END evidence network. Requests were also received informally from NCCMT colleagues, and sometimes suggested internally by NCCMT staff.

A weekly team meeting was implemented to assess progress on current reviews and assess capacity for new reviews, review the week’s schedule for review completion, assign staff tasks, and prioritize new questions and updates to previously completed reviews. Decisions as to which questions to accept were made through team discussion at the weekly team meeting and were based on several factors including: the urgency and relevance to Canada; in-house content expertise and capacity; and availability of evidence, determined by a preliminary scan of databases. Once accepted, an RR Lead is assigned. The Search Lead first looks to see if a recently completed review on the topic exists by scanning COVID-19 RR repositories and websites of organizations known to conduct rigorous syntheses. This step was introduced after we identified duplication of efforts between our team and others (Additional File 1). At first, we considered a synthesis with a search completed within the last week to be ‘up-to-date’. If another synthesis meeting this criterion was identified, we would not proceed with the question and informed the requestor of the other review. As the volume of evidence has grown, and the urgency of decision-making has slowed, as of May 2021 a search completed in the two months is considered ‘up-to-date’.

If a recent review is not available, the RR Lead and RES Coordinator meet to refine the question using PICO/PECO (Population, Intervention/Exposure, Comparison, Outcome) or PS (Population, Situation) format and identify inclusion/exclusion criteria and preliminary search terms. Unless the question is very clear or is an update to a previously completed review, the team will confirm the refined question and proposed criteria with the requestor. This usually involves narrowing the scope of the review to a question that is feasible to answer in the given time frame.

### Searching for evidence

Searching for evidence involves developing a search strategy and screening results for inclusion. The RR Lead, RES Coordinator, and RR Search Lead/Staff collaboratively develop a strategy for each question including databases to search, search terms and parameters for each database, and whether grey literature will be included. Our initial search strategy included 14 databases or websites, ten of which were developed specifically for COVID-19 (Table 2). The search may involve an advanced keyword string (e.g., The World Health Organization’s COVID-19 Global Literature on Coronavirus Disease) or a manual site scan (e.g., Public Health England’s completed reviews). Over time, the list of databases evolved as some collections were duplicative of others or were no longer updated frequently (e.g., Cochrane COVID Review bank), others were developed (e.g., L·OVE) and decisions to change to different databases were made to take search sophistication into account (e.g., switching from LitCovid to Medline) (Additional File 1). For searches where a very large number of results are identified that may not be feasible to screen, a very small number of results are identified, or the results appear largely irrelevant, a sample of search results is sent to the RR Lead to revise the search strategy; a health sciences librarian may also be contacted for guidance.

**Table 2:** NCCMT Rapid Evidence Service search strategy: COVID-19-relevant databases

Organization	Database	URL
Medline	Medline (OVID)*	https://www.wolterskluwer.com/en/solutions/ovid/ovid-medline-901
World Health Organization	Global literature on coronavirus disease*	https://search.bvsalud.org/global-literature-on-novel-coronavirus-2019-ncov/
McMaster University	McMaster PLUS™ COVID-19 Evidence Alerts*	https://plus.mcmaster.ca/COVID-19/
	McMaster Health Forum	https://www.mcmasterforum.org/
MedRxiv	Preprint Server for Health Sciences*	https://www.medrxiv.org/
Epistemonikos	COVID-19 Living Overview of the Evidence (L·OVE)*	https://app.iloveevidence.com/
University of York	Prospero Registry of Systematic Reviews	https://www.crd.york.ac.uk/prospero/
Cochrane	COVID Review Bank	https://covidreviews.cochrane.org/search/site
University of Oxford	Oxford COVID-19 Evidence Service: Current Questions Under Review	https://www.cebm.net/covid-19/current-questions-under-review/
NCCMT	COVID-19 Rapid Evidence Reviews	https://www.nccmt.ca/covid-19/covid-19-evidence-reviews
The University of Edinburgh	Uncover (USHER Network for COVID-19 Evidence Reviews)	https://www.ed.ac.uk/usher/uncover
Alberta Health Services	Catalogue of internally completed syntheses	https://www.albertahealthservices.ca/
CDC	Morbidity and Mortality Weekly Report	https://www.cdc.gov/mmwr/Novel_Coronavirus_Reports.html
Public Health England	Catalogue of internally completed syntheses	https://phe.koha-ptfs.co.uk/
*Denotes high yield database Abbreviations: CDC: Centers for Disease Control and Prevention; NCCMT: National Collaborating Centre for Methods and Tools *Note*: Other databases may be searched when relevant to the research question(s); a comprehensive list of databases, as well as additional information and descriptions, can be found in the Supplementary Materials.		

Searches are conducted in English; peer-reviewed, preprints, and non-peer reviewed reports are included. When titles and abstracts for non-English publications are available in English, and are sufficient to determine eligibility for inclusion, we use in-house expertise (French, Portuguese) or Google Translate. Depending on the question, we may consider a search for data from various public health jurisdictions (e.g., policy documents, regional surveillance data), to supplement scientific evidence or when this may enhance our ability to answer a question. For example, in a review on COVID-19’s role on substance use, overdoses, and substance-related deaths, we supplemented the search of scientific literature with available surveillance data across Canada [27].

Initially, most COVID-19-specific databases and repositories did not have functions to export all references into reference management software. To accommodate this, RR Search Staff entered potentially eligible studies, based on title and abstract screening, into an Excel spreadsheet for full text screening by the RR Lead. As the functionality for many databases evolved, we began to export references into Rayyan – an open access systematic review screening software [28] – for screening. Rayyan enhances efficiency by facilitating removal of duplicates and allowing simultaneous screening by multiple team members. We now use DistillerSR software (which requires a paid subscription) to facilitate deduplication of references, which is particularly helpful for review updates.

Title and abstract screening are done by a single reviewer, as per other RR guidelines [23, 29]. Full texts of potentially relevant articles are screened by the RR Lead to determine final inclusion. The DAISY AI feature in DistillerSR is used as a mechanism to ‘double check’ single reviewer screening, as it suggests references that may have been wrongly excluded.

For feasibility and timeliness, we prioritize guidelines and/or high-quality syntheses, when available. If a recent high-quality synthesis is available, we will consider excluding single studies or only including single studies after the last search date. To gauge quality, we look for whether a comprehensive search strategy is described and included evidence is critically appraised. If both criteria are met, we appraise the synthesis using AMSTAR 1 (A MeaSurement Tool to Assess systematic Reviews) [30]. A review that scores six or higher is deemed sufficient.

For questions where there were few systematic or rapid reviews or single studies identified, we may consider expert opinion or opinion-based guidance documents. These may include interim guidance documents from reputable organizations (such as the World Health Organization) that provide policy recommendations for a given aspect of pandemic response that was created through an expert panel for example. Although these are typically considered at the bottom of the hierarchy of evidence, given the novelty of the SARS-CoV-2 virus, and speed at which a response was required, these were sometimes considered the best available, or only available, evidence. These documents also underwent data extraction and critical appraisal as detailed below.

The RES first focused exclusively on new questions. As evidence continued to evolve, there was a need to update completed reviews and a strategy for identifying evidence that moved from preprint to publication stage was required. While some updated preprints were easily identified during searches, there were instances where substantial changes to the paper (e.g., authors, titles) made it difficult to identify when a preprint had been modified. While many entries into preprint servers are updated within four weeks of publication of peer-reviewed versions, this occurs inconsistently. We now systematically review previously included evidence to determine if it has been updated and if results have changed when completing an update. This includes a targeted search for: (1) updated versions to other included RRs (checking where the RR was published, searching via Google); (2) publication of preprint manuscripts and associated changes to data or interpretation (checking for duplicate first authors and/or titles in our search, checking the preprint server entry, searching via Google); and (3) updates to surveillance data or grey literature sources (checking original webpages). While this adds an additional step to the search process, it ensures we are including the most current evidence.

### Extracting data

We created a standard template to build the RR document for data extraction. Key information (question, search strategy, table of eligible studies) are added and sent to staff for data extraction. A single team member extracts data and summarizes key findings relevant to the specific research question; this is double checked by the RR Lead. Specific information depends on the research question, but typically includes study design, quality of included single studies (syntheses only), setting, population characteristics, interventions/exposure, and key outcomes. Any results that are not relevant to the research question are not extracted. Study limitations are noted to inform key findings and recommendations.

### Critically appraising the evidence

We critically appraise evidence using AMSTAR 1 for systematic reviews and Joanna Briggs Institute critical appraisal tools for other study designs [30, 31]. Some of our first RRs used the Health Evidence Quality Assessment tool [32]; we changed to AMSTAR 1 to contribute to a repository of critically appraised COVID-19 syntheses [33]. Critical appraisal is completed by one reviewer (internal staff, external contractor) and verified by a second. Conflicts are resolved through discussion or by the RES Coordinator. We assign an overall quality rating (strong, moderate, low) based on the total score. For example, the Joanna Briggs Institute tool for prevalence studies has a total of 9 items: ratings of 1–3 are assigned low quality, 4–6 moderate quality, and 7–9 high quality. Only the overall ratings are included in the RR; full critical appraisal is available upon request.

### “GRADE-ing” the evidence

In initial RRs, we reported on the number of studies of low, moderate, and high quality to report overall quality of evidence. But we were concerned that, although studies were appraised as high methodological quality, they were based on designs that had inherently high risk of bias (e.g., case reports, cross-sectional); thus, overall confidence in the evidence was low. In response, we adapted the GRADE approach [25, 34]: an assessment of the certainty in findings based on eight domains. In the GRADE approach, observational studies, for example, provide low quality evidence. This assessment can be further reduced based on: risk of bias; inconsistency in effects; indirectness of interventions/outcomes; imprecision in effect estimate; and publication bias [25]. The assessment can be upgraded based on a large effect, evidence of a dose-response relationship, and properly accounting for confounding. The overall certainty of the evidence (strong, moderate, low, very low) for each outcome is determined [25]. GRADE is completed by the RR Lead after reviewing the data extraction and results summaries from all included studies and is reviewed by the NCCMT Scientific Director and RES Scientific Lead.

### Synthesizing the evidence

Results are synthesized narratively due to variation in methodology and outcomes across included studies. Following data extraction, critical appraisal, and GRADE, the RR Lead completes the final synthesis for the Executive Summary. Early RR versions did not include an overall synthesis; this was added in response to requests from decision-makers for a high-level summary of key points, overview of evidence, and knowledge gaps, to be presented first. This revised layout more closely aligns with recommendations for communicating evidence to policymakers, including using a “graded entry” approach (1:3:25 page format), which allows users to access their preferred level of detail (e.g., from key points to full data) [35–37].

### Formatting and approving the final review

RRs are reviewed internally by the RR Lead, RES Scientific Lead, and NCCMT Scientific Director. For partnered RRs (e.g. a RR related to Indigenous health, partnered with the National Collaborating Centre for Indigenous Health [38]), partner organizations review the Executive Summary and results tables. Final formatting then ensures included evidence sources are appropriately cited and the document’s appearance conforms to the NCCMT’s style guide (Table 3).

**Table 3:** Structure of an NCCMT COVID-19 rapid review

Section	Details
Executive Summary (1–2 pages)	• Background of the topic and rationale for review • Research question(s) • Key take-away points • GRADE statements about certainty of evidence • Overview of evidence and knowledge gaps • (*For updates) “*What has changed in this version”
Methods	• Overview of search strategy (link to full strategy) • Date the search occurred • Inclusion and exclusion criteria • Process for data extraction, critical appraisal (i.e., which tools), and GRADE • (*For updates) “*What has changed in this version”
Findings	• GRADE Summary of Findings table • Data tables with summaries of each included study (fully referenced and linked at end of review) and their methodological quality
Abbreviations: GRADE; Grading of Recommendations, Assessment, Development and Evaluation; NCCMT: National Collaborating Centre for Methods and Tools	

### Disseminating the review

A tailored knowledge translation plan is developed for each RR depending on the topic and target audiences. When the review is requested by an organization, it is shared immediately upon completion. All RRs are freely available to download from the NCCMT website [39]. In September 2020, we created an RES email subscription, which notifies subscribers each time a new RR is posted. We alert our larger NCCMT subscriber-base (> 15,900 as of May 2021) by including new reviews and updates in our monthly newsletter. Reviews are included in monthly spotlights through the McMaster Health Forum and COVID-END [40]. We conduct targeted outreach via email to senior Canadian public health decision-makers and content-specific experts, as appropriate. We may reach out to media outlets via our institution’s public relations and communications department. Finally, we notify our social media followers via Twitter.

### Evaluating impact

From May 2020 to July 2021, the NCCMT’s RES team has answered more than 31 distinct questions and completed 32 updates to previously completed reviews (ranging from 1 to 16 updates per question) as new evidence emerged. Preliminary data from web analytics to assess engagement shows our RRs are accessed by all Canadian provinces and territories and 99 countries worldwide. Metrics collected from September 2020 to July 2021 indicate that each review is typically accessed 216 times within the first week, with wide variation in access across reviews. For example, our review on the role of schools and daycares in COVID-19 has been our mostly highly accessed [41]. Between May 2020 and April 2021, the living review has been updated 16 times, and has been viewed on our website over 7,700 times across 58 countries. The review has been cited and indexed in at least 79 sources, including key governmental and non-governmental reports and guidelines. This review has also been picked up by over 40 local, national, and international media outlets, and cited in other guidance documents [42]. Further evaluation of dissemination of this review indicates the international reach of the NCCMT’s reviews. Anecdotal feedback from both local and senior decision-makers within Canada reinforces that the RRs are helpful and informative to Canadian decision-makers. A more fulsome evaluation of the impact of the NCCMT’s RES on decision-making in Canada is planned, which will include a survey of senior decision-makers, key informant interviews, and a comprehensive analysis of web analytics and citation tracking. .

## Challenges, lessons learned, and limitations

A primary challenge to any RR is balance between speed and rigor. This issue is even more pronounced in the context of COVID-19 given the massive amount of data and the urgency with which evidence is needed to inform decisions. The streamlined approach of RRs (e.g., single reviewer screening) will always introduce some degree of bias, so it is important to establish and follow a transparent process. The evolving evidence landscape has necessitated many changes to our typical RR methodology; we anticipate further changes may be needed. For example, in addition to accepting and addressing new and emerging public health questions, we have committed to maintaining a living RR of the role of schools and daycares in COVID-19 transmission [41], given the ongoing importance of this question.

We are aware of other Canadian and international organizations conducting RRs on a number of topics related to COVID-19; many report adaptations to the RR process that are similar to ours [17, 23, 43–46]. For example, many include preprints and grey literature, when previously, only published sources were included [23]. Cochrane emphasizes involving stakeholders throughout the process to better tailor the RR for decision-making [17] and established a question identification and prioritization approach [45]. The Usher Network for COV(id) Evidence Reviews (UNCOVER) recommends against restricting to English-language, as a large volume of COVID-19 research has emerged from non-English-speaking countries [46]. All groups reiterate the importance of speed and critical appraisal, some adopting new software to achieve this [17, 23, 45, 46].

Although wide variations in RR methodology have been reported both in response to the COVID-19 pandemic, and in general [16], overall those conducting COVID-19 specific RRs appear to have distinct methodological features across groups. A 2015 scoping review of RR methods, of those that reported the duration of conduct of the RR, most were completed in 1–6 months [16]; while many COVID-19 RRs are produced more quickly. Within general rapid reviews, the most common approaches to streamlining include: presenting results as a narrative summary (78 %), limiting criteria by date (68 %), liming the search to published literature (24 %), and having one person extract data and a second person verify (23 %) [16].

From January 1-April 30, 2020, there were > 6,000 COVID-19-related manuscripts posted across preprint servers with > 250 preprints posted weekly [47]. While this rapid response is impressive, there is the potential that quick production of poor quality, non-peer-reviewed evidence may later be retracted or substantially alter its findings before publication [48]. Critical appraisal is therefore imperative, but also challenging as our team noted that methods often included minimal details. Like the NCCMT, many groups have recognized the need to assess the certainty in the findings and have incorporated GRADE [17].

Our organization is fortunate to have been well-prepared, in terms of staff expertise and funder support, to rapidly respond to public health needs in this time of crisis. Given specialized skills and a dedicated, nimble team, it was possible to pivot from previous workplans and use rigorous methods to develop RRs in a much faster timeframe than has been reported pre-pandemic (average 3.2 months [15], range 1–12 months [16]). However, we faced a number of challenges from both a human and financial resources perspective. While some reviews fell neatly within our planned time frames, some required greater resource allocation due to the number of eligible studies (e.g. a RR on transmission risk in acute care settings [49]). Although our team has expertise in searching, critical appraisal, and synthesis, we do not have content area expertise in all fields related to infectious diseases. For questions focused on basic science, laboratory, or mathematical modelling studies, we connected with modelling and infectious disease experts at McMaster Univesity and the National Collaborating Centres for Indigenous Health, Infectious Disease, and Environmental Health. Over time, we have shifted our response to these questions by either partnering with other oganizations with specific content expertise to support completion or recommending other organizations that could complete the review.

While many international RR groups focus specifically on clinical or treatment-related questions [50–52], few focus exclusively on public-health relevant topics. In our topic selection process, we regularly scan relevant websites and repositories to decrease the chance of duplication, but due to the time lag in agreeing to take on a question and having the final product available online, we are aware of instances where duplication of efforts has occurred. RRs may be considered outdated soon after completion due to the speed at which evidence is available. While we have integrated RR updates into our workflow, it is not possible to update all topics. An ongoing challenge is how to handle reviews that may no longer be based on the most recent and highest quality evidence. There is a need to combine forces and identify mechanisms for effective communication and sharing of resources to ensure that timely and rigorous reviews can be completed and shared amongst organizations to contribute to the global pandemic response. We are actively working on developing strategies to collaborate with provincial, national, and international organizations conducting public health relevant reviews to avoid duplication. Participation in COVID-END [26], funded by the Canadian Institutes of Health Research (CIHR) [53], is one strategy that helps to reduce duplication, as well as the NCCMT’s RR repository of ongoing and recently completed RRs related to COVID-19 [54].

### Future directions for the RES

Most of our efforts have been to conduct knowledge syntheses on priority public health questions and to broadly share findings with decision-makers. As the urgency to complete reviews has diminished somewhat, there is opportunity to expand our knowledge translation to diverse audiences and to identify new ways to support implementation of evidence into policy and program decisions. We also now seek to formally evaluate our process and its impact on public health decision-making.

## Conclusions

This overview provides a real-world example of how internationally accepted RR methods can be modified to meet the emergent needs of public health decision-makers in the unprecedented context of the COVID-19 pandemic. As countries around the world continue to grapple with ongoing issues including vaccine rollout, variants of concern, public distrust, fatigue with pandemic-related restrictions, and social and economic inequalities, there has never been a more important time to work collaboratively and in partnership with decision-makers to ensure the best available evidence is available to inform policy decisions and program planning.

## Supplementary Information

**Additional file 1.**

## Data Availability

Data sharing is not applicable to this article as no datasets were generated or analysed during the current study.

## Abbreviations

AMSTAR: A MeaSurement Tool to Assess systematic Reviews
CDC: Centers for Disease Control and Prevention
CIHR: Canadian Institutes of Health Research
COVID-19: Coronavirus Disease 2019
COVID-END: COVID-19 Evidence Network to support Decision-Makers
GRADE: Grading of Recommendations, Assessment, Development and Evaluation
NCCMT: National Collaborating Centre for Methods and Tools
NIHR: National Institute for Health Research
PHAC: Public Health Agency of Canada
PICO/PECO: Population, Intervention/Exposure, Comparison, Outcome
PS: Population, Situation
RES: Rapid Evidence Service
RR: Rapid Review
SARS: Severe Acute Respiratory Syndrome
UNCOVER: Usher Network for COV(id) Evidence Reviews
WHO: World Health Organization
