# Supplementary material for: Methods to support evidence-informed decision-making in the midst of COVID-19: creation and evolution of a rapid review service from the National Collaborating Centre for Methods and Tools
Source: BMC Med Res Methodol. 2021 Oct 27;21:231. doi: 10.1186/s12874-021-01436-1 (PMC8548700; doi:10.1186/s12874-021-01436-1)
Supplement: Supplementary file 1 — Additional file 1. [file 12874_2021_1436_MOESM1_ESM.docx]

**Additional File 1: A comprehensive list of databases from the National Collaborating Centre for Methods and Tools’s Rapid Evidence Service search strategy** (as of May 2021)

| **Database** | **Description & Rationale** | **Export function** | **Month added to search strategy** | **Month removed from search strategy** |
| --- | --- | --- | --- | --- |
| **High yield / robust databases** | | | | |
| Ovid [Medline](https://www.wolterskluwer.com/en/solutions/ovid/ovid-medline-901) | Subset of PubMed; can be searched using multiple key terms and controlled vocabulary | Y | March 2021 | N/A |
| World Health Organization’s [Global literature on coronavirus disease](https://search.bvsalud.org/global-literature-on-novel-coronavirus-2019-ncov/) | Indexes from databases that host peer-reviewed literature, as well as preprint servers; can be searched using multiple key terms | Y | April 2020 | N/A |
| [COVID-19 Evidence Alerts](https://plus.mcmaster.ca/COVID-19/Home) from McMaster PLUS™ | Indexes COVID-19 syntheses and single studies from MEDLINE; syntheses that meet inclusion criteria are critically appraised, and high-quality studies are easy to locate | Y | April 2020 | N/A |
| [MedRxiv preprint server](https://www.medrxiv.org/) | Preprint server that hosts medical, clinical, and health science studies that have not undergone peer review | Y | June 2020 | N/A |
| [COVID-19 Living Overview of the Evidence (L·OVE)](https://app.iloveevidence.com/loves/5e6fdb9669c00e4ac072701d) | Indexes from databases that host peer-reviewed literature, as well as preprint servers | Y | June 2020 | N/A |
| **Databases that host in-progress evidence** | | | | |
| [Prospero Registry of Systematic Reviews](https://www.crd.york.ac.uk/prospero/display_record.php?RecordID=193751) | Hosts in-progress syntheses submitted by research teams; continually updated | N | June 2020 | N/A |
| Cochrane Rapid Reviews [Question Bank](https://covidrapidreviews.cochrane.org/search/site) | Hosts studies that are both in-progress and not yet begun; list ceased to be updated (last update: Spring 2020) | N | April 2020 | June 2020 |
| Oxford COVID-19 Evidence Service: [Current Questions Under Review](https://www.cebm.net/covid-19/current-questions-under-review/) | Hosts studies that are both in-progress and not yet begun; no indication of whether questions were moving ahead; list ceased to be updated (last update: Spring 2020) | N | April 2020 | May 2020 |
| **Repositories that host internally completed studies** | | | | |
| National Collaborating Centre for Methods and Tools’ [COVID-19 Rapid Evidence Reviews](https://www.nccmt.ca/knowledge-repositories/covid-19-evidence-reviews) | Hosts completed and in-progress public health reviews from Canada and internationally; continually updated | N | May 2020 | N/A |
| [McMaster Health Forum](https://www.mcmasterforum.org/find-evidence/products/topic/?topic=All+health-+and+social-system+topics) | Lists internally completed social- and health-systems focused reviews; frequently updated | N | May 2020 | N/A |
| [Uncover (USHER Network for COVID-19 Evidence Reviews](https://www.ed.ac.uk/usher/uncover)) | Lists internally completed reviews on children & schools, ethnicity, facemasks, indoor & outdoor transmission, vaccines, and surveillance; updated semi-frequently | N | June 2020 | N/A |
| [Alberta Health Services](https://www.albertahealthservices.ca/) | Lists internally completed syntheses; updated semi-frequently | N | July 2020 | N/A |
| Centers for Disease Control and Prevention’s [Morbidity and Mortality Weekly Report](https://www.cdc.gov/mmwr/Novel_Coronavirus_Reports.html) | Lists single studies, including prevalence data and case reports; updated weekly | N | August 2020 | N/A |
| [Public Health England](https://phe.koha-ptfs.co.uk/) | Lists internally completed syntheses; updated semi-frequently | N | November 2020 | N/A |
| **Databases searched only when relevant to the research question** | | | | |
| [Trip Medical Database](https://www.tripdatabase.com/) | Hosts guidelines and systematic reviews; only applicable for reviews that have a policy or guideline focus | Y^1^ | April 2020 | N/A |
| [Covid Mental Health (CMH) Initiative: Research](https://covid19mentalhealthresearch.ca/research/) | Hosts Government of Canada syntheses on COVID-19 and mental health and addictions; only applicable for rapid reviews that have a mental health focus | N | June 2020 | N/A |
| [PsyArXiv](https://psyarxiv.com/) | Preprint server that hosts psychological sciences studies that have not undergone peer review; only applicable for rapid reviews that have a psychological science focus | N | September 2020 | N/A |
| [PsycINFO](https://search.proquest.com/psycinfo/advanced) | Hosts evidence in psychology and related disciplines; only applicable for rapid reviews that have a psychological science focus | Y | July 2020 | N/A |
| [BC Centers for Disease Control](http://covid-19.bccdc.ca/) | Includes prevalence data and public guidelines; only applicable for rapid reviews with a prevalence or jurisdictional focus | N | June 2020 | N/A |
| [Institut national de santé publique du Québec (INSPQ)](https://www.inspq.qc.ca/) | Hosts internally published prevalence reports, syntheses, and expert opinion pieces; includes guidelines for select settings; only applicable for rapid reviews with a prevalence focus or a location-specific focus | N | June 2020 | N/A |
| [Institute national d’excellence en santé et en services sociaux (INESSS)](https://www.inesss.qc.ca/covid-19/services-sociaux.html) | Hosts internally published reviews on pertaining to COVID-19 and youth, social services and equity; only applicable for rapid reviews that have a youth, social services, or equity focus | N | June 2020 | N/A |
| [National Collaborating Centre for Determinants of Health](http://nccdh.ca/our-work/covid-19)’s [Equity-informed Responses to COVID-19](http://nccdh.ca/our-work/covid-19) | Hosts evidence pertaining to determinants of health and COVID-19; only applicable for rapid reviews that have an equity focus | N | June 2020 | N/A |
| [National Collaborating Centre for Environmental Health](https://ncceh.ca/environmental-health-in-canada/health-agency-projects/environmental-health-resources-covid-19)’s [Environmental Health Resources for the COVID-19 Pandemic](https://ncceh.ca/environmental-health-in-canada/health-agency-projects/environmental-health-resources-covid-19) | Hosts evidence pertaining to environmental health and COVID-19; only applicable for rapid reviews that have an environmental health focus | N | June 2020 | N/A |
| [National Collaborating Centre for Health Public Policy](http://www.ncchpp.ca/en/)’s [Public Health Ethics and COVID-19](https://www.nccih.ca/485/NCCIH_in_the_News.nccih?id=450) | Hosts evidence pertaining to policy and COVID-19; only applicable for rapid reviews that have a policy focus | N | June 2020 | N/A |
| National Collaborating Centre for Infectious Diseases’ [News](https://nccid.ca/) | Hosts evidence pertaining to infection control and COVID-19; only applicable for rapid reviews that have an infectious disease focus | N | June 2020 | N/A |
| [National Collaborating Centre for Indigenous Health](https://www.nccih.ca/485/NCCIH_in_the_News.nccih?id=450)’s [Updates on COVID-19](https://www.nccih.ca/485/NCCIH_in_the_News.nccih?id=450) | Hosts evidence pertaining to indigenous health and COVID-19; only applicable for rapid reviews that have an indigenous health focus | N | June 2020 | N/A |
| [Sociological Abstracts](https://www-proquest-com.libaccess.lib.mcmaster.ca/sociologicalabstracts/advanced) database | Hosts evidence pertaining to the social and behavioural sciences; only applicable to reviews that have a social science focus | Y | March 2021 | N/A |
| [Cumulative Index to Nursing and Allied Health Literature (CINAHL)](https://www.ebsco.com/products/research-databases/cinahl-complete) | Hosts evidence pertaining to nursing and health sciences; only applicable to reviews that have a health science and practice focus | Y | March 2021 | N/A |
| [Educational Resources Information Centre (ERIC)](https://www.ebsco.com/products/research-databases/eric) | Hosts evidence pertaining to education; only applicable to reviews that have an education focus | Y | March 2021 | N/A |
| [Scopus](https://www.scopus.com/home.uri) database | Hosts evidence pertaining to science, medicine, social sciences and humanities; only applicable to reviews with a social science focus | Y | May 2021 | N/A |
| [PubMed](https://pubmed.ncbi.nlm.nih.gov/) database | Hosts biomedical and life sciences literature; only applicable for rapid reviews that include events prior to the COVID-19 pandemic | Y | May 2020 | March 2021 |
| [EMBASE](https://www.embase.com/login) database | Hosts pharmacology, toxicology, and biomedical evidence; only applicable for rapid reviews that include events prior to the COVID-19 pandemic | Y | May 2020 | N/A |
| **Databases we ceased searching** | | | | |
| Pubmed’s curated COVID-19 literature hub: [LitCovid](https://www.ncbi.nlm.nih.gov/research/coronavirus/) | Indexes from PubMed; redundant because citations are retrieved through the Medline search | Y | April 2020 | May 2021 |
| [Public Health+](https://www.nccmt.ca/knowledge-repositories/public-health-plus) | Database of pre-appraised public health studies, indexed from McMaster+; redundant because reviews are captured as part of the McMaster+ search | N | April 2020 | October 2020 |
| [Newfoundland & Labrador Centre for Applied Health Research (NLCAHR)](https://www.nlcahr.mun.ca/) | Hosts internally completed rapid reviews on COVID-19; redundant because all reviews are captured within the National Collaborating Centre for Methods and Tools’ COVID-19 Rapid Evidence Reviews search | N | June 2020 | August 2020 |
| Joanna Briggs Institute [COVID-19 Special Collection](https://joannabriggs.org/ebp/covid-19) | Hosts infection prevention and control guidelines; database ceased to update | N | April 2020 | September 2020 |
| [CovidReview](https://covidreview.ca/) | Hosts high-quality, peer-reviewed literature on COVID-19; redundant because the database indexes from PubMed, which is captured as part of the LitCovid search | N | April 2020 | May 2020 |
| [Oxford COVID-19 Evidence Service](https://www.cebm.net/oxford-covid-19-evidence-service/) | Hosts prevalence data, jurisdictional evidence, and expert opinion pieces | N | April 2020 | N/A^2^ |
| [Epistemonikos](https://www.epistemonikos.org/en/) | Database of health-related systematic review; redundant because all Epistemonikos evidence is captured in L-OVE | Y | April 2020 | May 2020 |
| [Guidelines International Network](https://g-i-n.net/home)  [(G-I-N)](https://g-i-n.net/home) | Includes list of locations that host evidence; does not host evidence | N | April 2020 | May 2020 |
| ^1^ Trip has an export function that is only available with a paid subscription. ^2^ We stopped searching the Oxford COVID-19 Evidence Service from July-October due to limited new data but have begun searching here again as of November 2020. | | | | |
